# Anorectal Malformation Associated with Small and Large Bowel Atresias: A Rare Association

**Published:** 2012-10-01

**Authors:** Sujay Maitra, Ruchirendu Sarkar

**Affiliations:** Department of Pediatric Surgery, NRS Medical College, Kolkata

**Dear Sir**

Anorectal malformation (ARM) is one of frequent neonatal surgical problem managed in pediatric surgical units. Gastrointestinal malformations are associated in approximately 5% cases of ARM. Some of the common associations are oesophageal atresia and trachea oesophageal fistula, duodenal atresia, hirschsprung`s disease, pouch colon and neuronal intestinal dysplasia [1]. Association of ARM with either colonic atresia or jejuno-ileal atresia has rarely been reported in literature [2-5]. Here we report a case of ARM associated with both colonic and multiple jejuno-ileal atresias.

A 2-day-old male neonate, weighing 1.1Kg, premature (33 wks); first born child of non-consanguineous parents presented to our paediatric emergency with complaint of absence of anus and bilious vomiting. There was no history of ingestion of any drug apart from those prescribed for pregnancy. At presentation the neonate was mildly dehydrated. Abdomen was scaphoid with fullness of upper epigastrium. Anus was absent with poorly developed gluteal muscle suggesting high ARM. Spine and sacrum was normal. Straight x-ray showed few gas levels in upper abdomen without any visible gas shadow in the lower abdomen suggestive of complete upper gastrointestinal obstruction in addition to ARM. The patient was resuscitated with intravenous fluid, electrolyte imbalance was corrected and surgery was performed under antibiotic coverage. Multiple jejuno-ileal atresias (eight in number, mixed in nature, predominantly mucosal webs) and a colonic atresia at the level of left colic flexure was identified (Fig. 1). Sigmoid colon up to rectosigmoid junction was found as microcolon. Jejuno-ileal atretic segments were excised and intestinal continuity was restored by end to end anastomosis. Postoperative measurement showed twenty five centimetres of small bowel as residue. Colonic atretic segment was exteriorised as end colostomy. Residual sigmoid colon was opened as a mucous fistula. Post operatively there was no anastomotic leak but the child sustained dehydration, weight loss and nutritional deficiency for which partial parenteral nutrition has been given as supplement. The patient is on our follow up for the management of short bowel syndrome.

**Figure:**
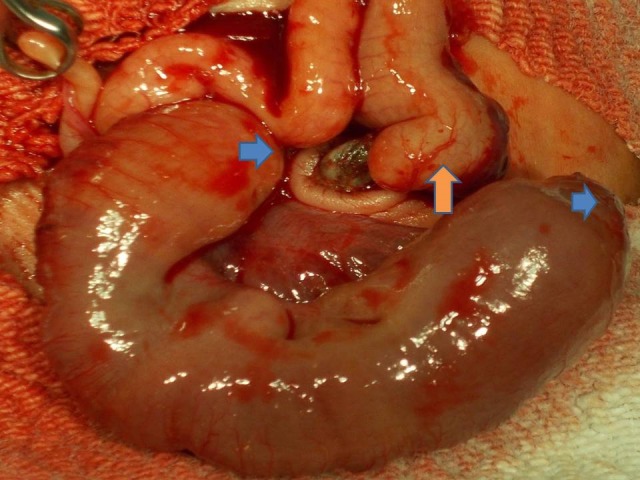
Figure 1: Multiple small bowel (blue arrow) atresias and a large bowel (salmon arrow) atresia.

ARM is a common congenital malformation with an incidence of 1 in 1862 in eastern India [1]. Many types of gastrointestinal malformations have been described with ARM. Oesophageal atresia is the commonest gastro-intestinal association, found in approximately 10% of the patients. Duodenal atresia occurs in 2-3% of ARM patients [1]. Jejuno-ileal atresias have previously been described in association with ARM [2]. Similarly colonic atresias have also been reported in ARM patients in three patients [3-5]. Combined jejuno-ileal and colonic atresias have never been reported in association with ARM (to the best of authors' knowledge). Role of vascular accidents in aetio-pathogenesis of atresia may have some implications in the migration of ano-rectum in associated ARM cases. VACTERL association (vertebral, anorectal, cardiac, trachea-esophageal, renal, and limb) has been found in 10-20% of patients with ARM. 

Etiopathogenesis of atresia points towards vascular accidents much later in the embryological time-frame. Prematurity has probably some relationship to immaturity of blood vessels leading to vascular insult to the developing gut, giving rise to atresias. ARM patients presenting with drooling of saliva, bilious or non-bilious vomiting in presence of non distended abdomen must give rise to a suspicion of associated atresia in the mind of the attending surgeon [2-4]. Though rare, the association of atresias with ARM must be kept in mind during colostomy formation. Atresias in any form, if missed, will add to the morbidity and even mortality to the surgically curable disease ARM.

## Footnotes

**Source of Support:** Nil

**Conflict of Interest:** None
